# Nicotine-induced immune escape mechanisms in lung adenocarcinoma: ceRNA network toxicology, and molecular dynamics simulations

**DOI:** 10.7717/peerj.21103

**Published:** 2026-05-04

**Authors:** Yijing Zhong, Quhuan Li, Hailin Li, Xingyu Liu, Xiaomeng Chen, Jiao Li, Yu Zhang, Ruiquan Xu

**Affiliations:** 1 School of Biology and Biological Engineering, South China University of Technology, Guangzhou, China; 2 Gannan Medical University, Ganzhou, China; 3 The First Clinical School of Gannan Medical University, Ganzhou, China; 4 First Affiliated Hospital of Gannan Medical University, Ganzhou, China

**Keywords:** Lung adenocarcinoma, Nicotine, Network toxicology, Molecular docking, Molecular dynamics simulations

## Abstract

Lung adenocarcinoma (LUAD), the most common subtype of lung cancer and a leading cause of cancer-related deaths, is strongly associated with cigarette smoking and nicotine exposure. However, the molecular mechanisms underlying nicotine-induced LUAD remain unclear. This study employed an integrative approach combining network toxicology, competing endogenous RNA (ceRNA) analysis, molecular docking, and dynamics simulations to investigate nicotine’s role in LUAD. Network toxicology identified 81 potential nicotine targets, with 12 core targets showing significant differential expression in LUAD. Functional enrichment suggested involvement of immune escape, inflammation, and cell death. A ceRNA network highlighted microRNA-101 and microRNA-155-5p as key regulators. Molecular docking and dynamics simulations demonstrated stable binding between nicotine and core targets. These findings reveal toxicological mechanisms driving LUAD and offer potential therapeutic targets.

## Introduction

Lung cancer remains the leading cause of cancer-related mortality worldwide, with lung adenocarcinoma (LUAD) being the most prevalent histological subtype of non-small cell lung cancer (NSCLC) (Li et al., 2024; Wei et al., 2024). While cigarette smoke contains numerous carcinogens, nicotine, as the primary addictive component, plays a critical but often underestimated role in tumor progression. Although not a direct carcinogen, accumulating evidence suggests that nicotine promotes proliferation, angiogenesis, and therapy resistance in LUAD through the activation of nicotinic acetylcholine receptors (nAChRs) and downstream signaling cascades, including the PI3K/AKT and MAPK pathways (Huang et al., 2012; Murphy, 2021; Wang et al., 2020).

Despite these findings, a significant knowledge gap remains in our understanding of nicotine-induced LUAD. Most existing studies have focused on isolated receptors or single signaling pathways, failing to capture the systemic complexity of nicotine toxicity. Nicotine exposure triggers a multi-layered molecular response involving intricate interactions between proteins, genes, and non-coding RNAs. The lack of a comprehensive, network-level analysis limits our ability to identify core therapeutic targets and understand how nicotine exposure intensity correlates with molecular dysregulation in clinical settings.

To address this challenge, this study adopts an integrated network toxicology framework combined with clinical validation to systematically decode the mechanism of nicotine in LUAD (Zhang et al., 2015). We constructed a protein–protein interaction (PPI) network and utilized machine learning algorithms to screen for core targets critical to nicotine-associated LUAD progression. Beyond static network analysis, we incorporated competing endogenous RNA (ceRNA) network construction to uncover post-transcriptional regulatory mechanisms. Furthermore, we validated these targets using TCGA clinical data to assess expression trends across smoking exposure gradients and employed molecular docking and molecular dynamics (MD) simulations to suggest the physical stability of toxin–target interactions (Cheng et al., 2025; Zhang et al., 2025). This study provides a systematic perspective on the molecular landscape of nicotine toxicity, identifying robust biomarkers and potential therapeutic strategies for nicotine-associated lung adenocarcinoma.

## Materials & Methods

### Causal association and toxicity assessment

To investigate the causal relationships of smoking and nicotine with LUAD, Mendelian Randomization (MR) was performed *via* the TwoSampleMR package (v0.5.7) adhering to relevance, independence, and exclusion restriction assumptions. Instrumental variables (IVs) were rigorously screened (*P* < 1 × 10^−6^, *r*^2^ < 0.001 within a 10 kb window) and validated for strength (F-statistics > 10). Causal estimates were primarily derived using the Inverse Variance Weighted (IVW) method, corroborated by MR-Egger and Weighted Median approaches. Robustness was ensured through sensitivity analyses, including Cochran’s Q test for heterogeneity, MR-Egger intercept for horizontal pleiotropy, and leave-one-out cross-validation.

Further analysis of network toxicological effects of nicotine, the main addictive component of cigarettes, on LUAD is needed, considering the potential impact of smoking on LUAD. To evaluate the toxicity of nicotine, the simplified molecular input line entry system (SMILES) sequence was retrieved from the PubChem database (https://pubchem.ncbi.nlm.nih.gov/) and subsequently input into the ProTox 3.0 (https://tox.charite.de/protox3/index.php?site=home) and ADMETlab 3.0 (https://admetlab3.scbdd.com/server/screening) databases (accessed in December 2024), for toxicity prediction, with higher probabilities indicating greater toxicity risks.

### Collection of potential targets for nicotine

The molecular structure of nicotine was retrieved from the PubChem database. Potential nicotine targets were identified using the Superpred (https://prediction.charite.de/index.php), STITCH 5.0 (http://stitch.embl.de/) and comparative toxicogenomics database (CTD) (https://ctdbase.org/) databases (accessed in December 2024), which were restricted to *homo sapiens* to ensure biological relevance. The reference count for the selection criteria for targets from the CTD databases was set to >1 to ensure prediction reliability. Nicotine targets from the three databases were compiled and duplicate entries were removed. The final set of targets was standardized using reviewed data for *homo sapiens* from the UniProt database (accessed in December 2024, https://www.uniprot.org/), ensuring the accuracy and consistency of the gene data.

### Identification of core targets and PPI network construction

Potential disease targets were systematically retrieved from the GeneCards 5.25 (https://www.genecards.org/) and OMIM (https://omim.org/) databases (accessed in December 2024), using the keyword “Lung Adenocarcinoma”. To ensure data reliability, GeneCards targets were filtered based on a relevance score ≥ mean value (Zhuang et al., 2023), merged with OMIM entries, and deduplicated. The intersection of these LUAD-related targets and the identified nicotine targets was visualized using a Venn diagram to pinpoint key overlapping candidates. To reconstruct the interaction landscape, these overlapping targets were mapped to a protein–protein interaction (PPI) network using the STRING 12.0 database (accessed in December 2024, http://string-db.org), with parameters set to Homo sapiens and a minimum interaction score of 0.4. The network was visualized in Cytoscape 3.10.0, and significant functional modules were analyzed using the MCODE plugin. Finally, core targets were rigorously identified based on topological centrality, defined by the mean Degree (≥54) and MCODE score (>28) (Zhuang et al., 2023).

### Expression profiles and construction of diagnostic models for core targets

RNA sequencing (RNA-seq) data from LUAD cohorts were obtained from The Cancer Genome Atlas (TCGA; accessed in January 2025, https://portal.gdc.cancer.gov/) and normalized to transcripts per million (TPM) format. Differentially expressed genes (DEGs) between nicotine-exposed and non-exposed samples were identified using the limma package (—log2FC— > 1, *P* < 0.05). A diagnostic model was constructed using Least Absolute Shrinkage and Selection Operator (LASSO) regression, implemented with the glmnet package in R, to select key predictive genes and avoid overfitting. The dataset was randomly divided into training (80%) and test (20%) sets. LASSO coefficients were visualized and a tenfold cross-validation was performed to optimize the model. The area under the receiver operating characteristic (ROC) curve served as the primary metric for assessing model effectiveness. Coefficient path plots and cross-validation error plots were created to illustrate the feature selection and model stability.

### GO and KEGG enrichment analysis

The core targets identified in the PPI network were further analyzed for their biological functions and pathways using the DAVID database (accessed in January 2025, https://davidbioinformatics.nih.gov/). GO analysis was performed with *homo sapiens* as the species (*P* < 0.05) to identify significant biological processes (BP), molecular functions (MF), and cellular components (CC). The results were presented as bar charts. Additionally, KEGG pathway enrichment analysis was conducted to determine pathways associated with the core targets. KEGG results were visualized using a circle chart and analysis outcomes were further processed using ChiPlot (accessed in January 2025, https://chiplot.online/) for KEGG and GO pathway visualization.

### GeneMANIA-based functional association network analysis

Function-related targets of the core genes were predicted using the GeneMANIA database (accessed in January 2025, https://genemania.org/). The GMFA network approach identified 20 functionally relevant genes with gene co-expression, genetic interactions, and physical interactions for each of the core targets, and highlighted the genes that exhibited strong network connectivity. Subsequently, functionally related targets were combined with core targets to create a GMFA-enhanced database (GMFA-ED) for nicotine-associated LUAD. In particular, we focused on differentially and diagnostically expressed targets and their GMFA targets.

The GMFA-ED dataset was used for GO and KEGG pathway enrichment analyses. Important pathways in each GO category (BP, MF, and CC) and KEGG pathway were visualized using the ChiPlot database.

### Gene set variation enrichment analysis of GMFA-ED

GSVA was performed on the GMFA-ED dataset to assess the pathway activity in nicotine-induced LUAD samples. After the removal of duplicate genes, RNA-seq expression data were normalized using pathway gene sets obtained from the MsigDB database (accessed in February 2025, https://www.gsea-msigdb.org/gsea/msigdb) using the “h.all.v2024.1. Hs. symbols.gmt” collection. Gene sets were screened using the GSVA R package, with a minimum size = 10 and maximum size = 500. GSVA scores were combined with subtype information using the limma package. Linear modeling analysis was performed to identify differentially active pathways for —logFC— > 0.1 and *P* < 0.05.

### Competing endogenous RNA network construction

To construct the ceRNA network, we first used the miRDB database (accessed in February 2025, https://mirdb.org/mirdb/index.html) to identify potential microRNA (miRNA) targets among differentially and diagnostically expressed genes. To ensure high predictive reliability, we selected interactions with target scores ≥ 90 (Chen & Wang, 2020). We used the Encyclopedia of RNA Interactomes (ENCORI) database (accessed in February 2025, https://rnasysu.com/encori/) to screen for miRNAs, download, and screen for lncRNA interactions. Identified miRNA–mRNA and miRNA–lncRNA interactions were collated into two separate Excel files: one detailing the case relationships and the other describing the case types. Finally, processed data were imported into Cytoscape version 3.10.0 for network visualization.

### Molecular docking

Molecular docking was performed to investigate the binding interactions between nicotine and LUAD-related target proteins. Nicotine was selected as the docking ligand, with its three-dimensional structure obtained from the PubChem database and prepared by adding polar hydrogen and Gasteiger charges before conversion into PDBQT format (AutoDock Vina 1.5.7; (Forli et al., 2016). High-resolution human protein structures were downloaded from the Research Collaboration for Structural Bioinformatics Protein Data Bank (RCSB PDB; accessed in February 2025, https://www.rcsb.org/search). Protein preparation involved removing water molecules, filling in missing residues, adding hydrogen atoms. Crucially, binding pockets were defined based on the spatial coordinates of the original co-crystallized ligands within the PDB structures to ensure accurate active site targeting. Docking simulations were executed using AutoDock Vina with a standard exhaustiveness parameter of eight (Agarwal & Smith, 2023), a setting validated for optimal pose accuracy in standard search spaces. Specific grid box dimensions and center coordinates for each target are detailed in Table S17. The quality of molecular interactions was evaluated based on the binding affinity scores (kcal/mol), where lower binding energies indicate more stable ligand-protein complexes.

### Molecular dynamics simulations

Molecular dynamics (MD) simulations of the CASP3 protein complexes with nicotine ligands were performed using NAMD 2.14 software (Phillips et al., 2020). The CHARMM36 force field was selected for both the proteins and small molecules (Yu & Klauda, 2020). The transferable intermolecular potential with three points (TIP3P) water model was used to solvate the protein–ligand system within a periodic boundary water box. To mimic physiological conditions, the system was neutralized with 150 mM NaCl. Prior to the production run, energy minimization and equilibration of the system were carried out through the following steps: (1) Positional restraints were applied to protein, whereas water molecules and small-molecule inhibitors underwent energy minimization for 15,000 steps. The primary goal was to optimize the relative positions of proteins, inhibitors, and water molecules. (2) Positional restraints were applied to small molecules, whereas the protein and water molecules underwent energy minimization for 15,000 steps. (3) Positional restraints were applied to protein backbone atoms and the side chains of the amino acids were optimized through 15,000 steps of energy minimization. (4) All atoms underwent 15,000 energy-minimization steps to optimize the entire system. Energy-minimized systems were gradually heated from 0 to 310 K over 0.1 ns, followed by equilibration for 5 ns under constant pressure and temperature control. The temperature was maintained at 310 K using Langevin dynamics and the pressure was maintained at 1 atm using the Langevin piston method. Unrestrained MD simulations were performed thrice for each equilibrated system, with each run lasting 50 ns. Finally, we analyzed the MD trajectories for root-mean-square deviation (RMSD; Sargsyan, Grauffel & Lim, 2017), radius of gyration (Rg; Yanao et al., 2007), solvent-inaccessible surface area (buried-SASA; Wang & Hou, 2012), and the number of hydrogen bonds between protein and ligands. The residue interaction index (RII) was calculated from interactions at the binding interface. Hydrogen bonds were identified using the following criteria: a donor–acceptor distance of less than 0.35 nm and donor–hydrogen–acceptor angle <30°.

To further elucidate the dynamic behavior and structural evolution of nicotine in the system, we analyzed its root-mean-square fluctuation (RMSF), RMSD, Rg, and dihedral angle variations. This multi-faceted approach provides comprehensive insights into the molecular motion and structural stability of nicotine during the simulation. This study utilized VMD 1.9.3 (Humphrey, Dalke & Schulten, 1996) and immunohistochemical (IHC) images from the Human Protein Atlas 25.0 (HPA; accessed in October 2025, https://www.proteinatlas.org/) database to examine the expression of key nicotine target proteins (Antibody_CAB000091) in normal lung tissues and lung adenocarcinoma, thereby providing a basis for further mechanistic investigation.

## Results

### Causal association and toxicity assessment

MR analysis demonstrated a significant bidirectional causal relationship of smoking and nicotine with LUAD based on the IVW method (Figs. S1–S2 and Table S1). Following this, we profiled the toxicity of nicotine. ProTox 3.0 predicted a lethal dose (LD50) of 3 mg/kg (Class 1), while ADMETlab 3.0 profiling indicated broad organ toxicity, with notably high probability scores (>0.77) for respiratory, hepatic, and nephrotoxicity. These data underscore the high toxicological burden of nicotine on the respiratory system and specific organs.

### Identification of core molecular targets in nicotine-induced LUAD

**Figure 1:**
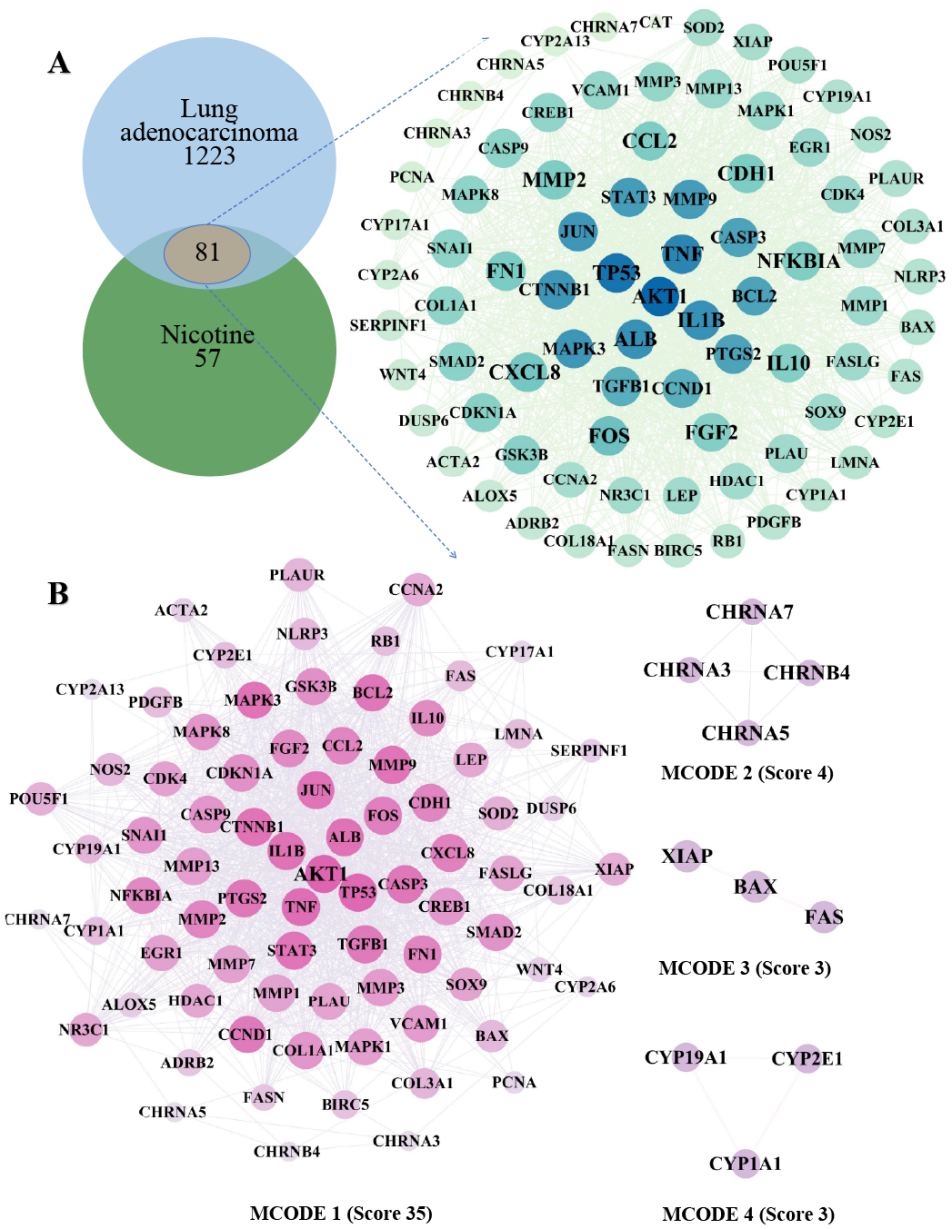
Protein–protein interaction (PPI) network diagram. (A) The PPI network graph consisting of intersecting goals consists of 80 nodes and 1,380 edges. Each node represents a target. The size of a node is positively correlated with its modularity score. The larger the node is, the greater is the degree of modularity within the network. The number of edges represents the frequency of interactions between targets. (B) PPI network diagram after modularity scoring. Following the modularity scoring analysis, the network was reduced to 79 nodes and 1,379 edges, highlighting the most central targets. The size of the circles reflects the modularity score, with larger circles indicating higher scores. Edges represent interactions between targets.

We employed a multi-database mining strategy to map the interaction landscape. By intersecting 138 nicotine-associated targets with 1,304 LUAD-related targets, we identified 81 overlapping genes serving as potential key mediators (Fig. 1A and Tables S2–S7). GeneCards database, with a correlation score greater than the average. Topological analysis of the PPI network constructed from these targets revealed a highly interconnected module (Table S8). MCODE clustering identified 12 core hub genes with high connectivity (Degree ≥ 54 and MCODE score > 28), including threonine-protein kinase (AKT1), Jun proto-oncogene, AP-1 transcription factor subunit (JUN), albumin (ALB), tumor protein p53 (TP53), signal transducer and activator of transcription 3 (STAT3), caspase 3 (CASP3), interleukin 1 Beta (IL1B), transforming growth factor beta 1 (TGFB1), Fos proto-oncogene, AP-1 transcription factor subunit (FOS), tumor necrosis factor (TNF), prostaglandin-endoperoxide synthase 2 (PTGS2), and catenin beta 1 (CTNNB1) (Fig. 1B). These hubs likely represent the central signaling architecture driving nicotine-induced pathogenesis in LUAD.

### Diagnostic validation and expression dysregulation of core targets

**Figure 2:**
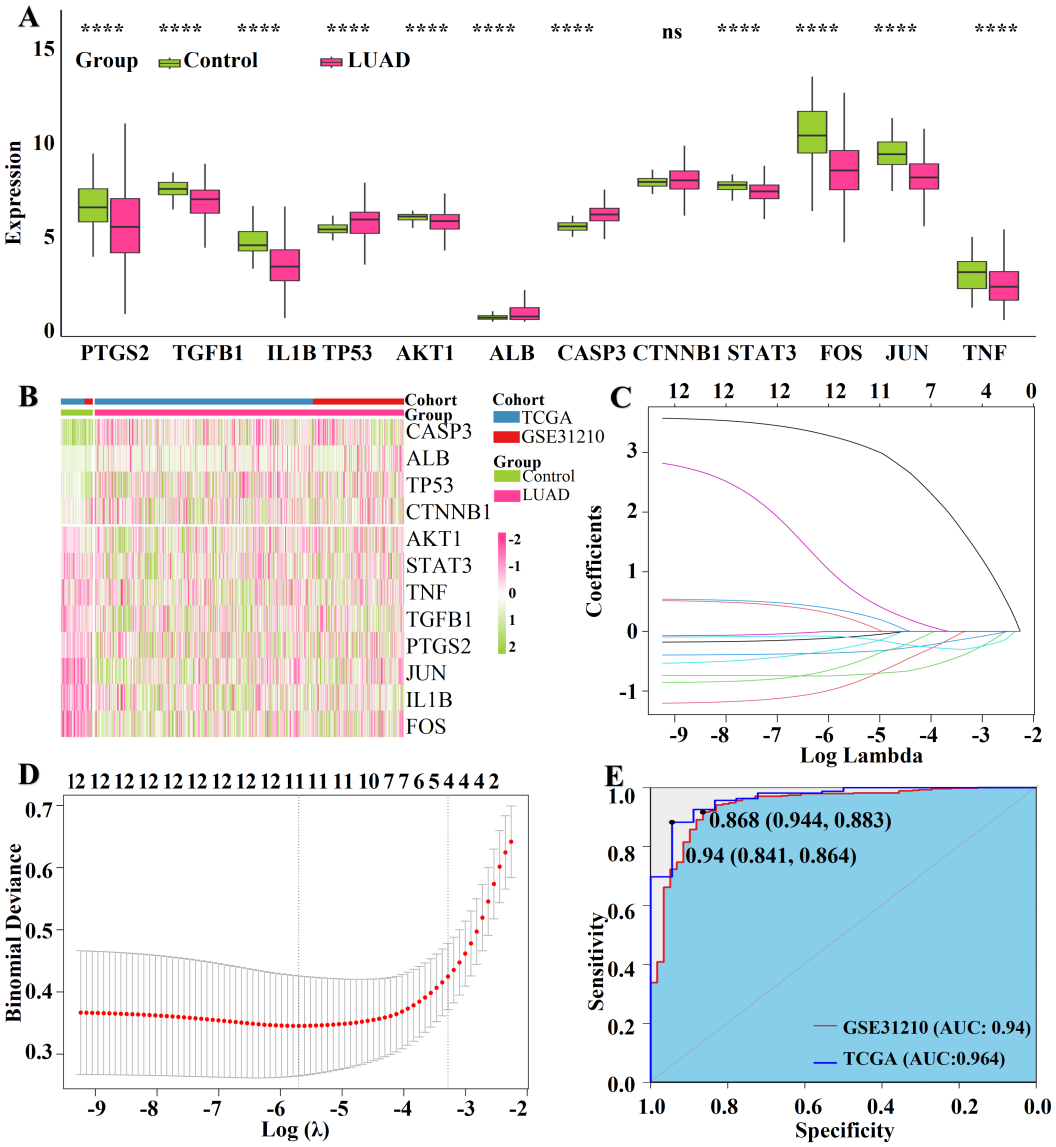
Core target expression analysis and diagnostic model development. (A) Differential expression of 12 core genes in LUAD *versus* normal tissues. (B) Heatmap of the 12 core genes. (C) Least Absolute Shrinkage and Selection Operator (LASSO) regression of the 12 hub genes calculated by MCODE. (D) LASSO regression parameter selection using cross-validation. (E) Receiver operating characteristic curves for the 12 key targets.

To assess diagnostic potential, we analyzed expression profiles in 541 LUAD samples (stratified into 429 smokers and 75 lifelong non-smokers) and 59 normal controls from the TCGA cohort. Tumor tissues exhibited significant upregulation of ALB, TP53, and CASP3, contrasting with the downregulation of inflammatory mediators such as AKT1, IL1B, TGFB1, and TNF (Figs. 2A–2B), suggesting a complex perturbation of immune and survival pathways. To evaluate clinical utility, we constructed a LASSO diagnostic model, with coefficient paths (Fig. 2C) and binomial deviance plots (Fig. 2D) confirming feature stability. The model demonstrated exceptional discriminatory power in the training cohort (TCGA, AUC = 0.964). Crucially, this robustness was confirmed in the independent external cohort GSE31210 (Figs. S3A–S3C), where the model maintained high predictive accuracy (AUC = 0.94; Fig. 2E). Specifically, CASP3 was identified as a positive risk factor, while IL1B, FOS, and JUN were negatively associated with disease risk, underscoring their potential as stable diagnostic biomarkers across diverse populations. Furthermore, to investigate whether these biomarkers might reflect nicotine-specific pathology rather than general tumorigenesis, we evaluated their response to exposure. Core targets demonstrated significant differential expression in nicotine-exposed groups (Fig. S3D), with CASP3 exhibiting a pronounced dose-dependent upregulation (Figs. S3E–S3F) and significant correlation with nAChR regulators (Fig. S3G). This multi-level validation highlights their potential as promising, mechanism-anchored diagnostic biomarkers.

### GO and KEGG enrichment analysis of core targets

**Figure 3:**
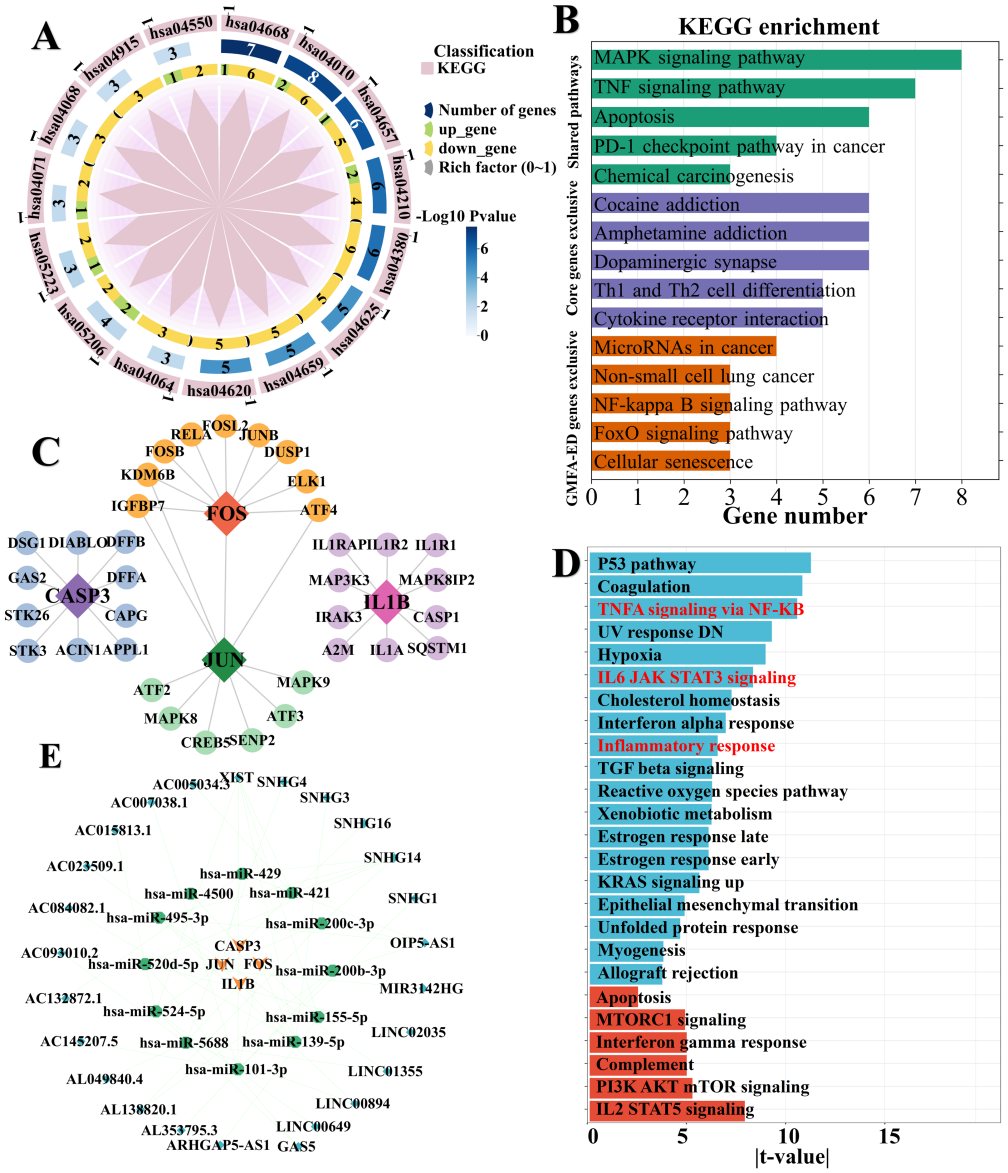
Expansion analysis of core genes. (A) 15 KEGG enrichment pathways for 12 core targets. The *P*-value represents the statistical significance of the enrichment analysis, where a smaller *P*-value indicates a more significant enrichment of the pathway. (B) Comparative KEGG analysis between the 12 core targets and the expanded gene network. (C) Four diagnostically significant genes and their GMFA genes. Diamonds mark the four differentially expressed diagnostic genes and circles indicate functionally related genes. (D) Bar plot of GSVA enrichment analysis for the 32 signaling pathways associated with nicotine-induced LUAD. Blue bars represent negative correlations, whereas red bars indicate positive correlations. (E) ceRNA regulatory network. The green hexagons represent key miRNA regulators; and blue diamonds represent upstream lncRNAs. Edges indicate experimentally validated or high-confidence predicted interactions within the lncRNA–miRNA–mRNA axes.

To explore the biological functions of these 12 core targets and the signaling pathways in which they are involved, we performed GO and KEGG enrichment analyses on 12 potential key targets in the DAVID database. After filtration, KEGG pathway analysis identified critical signaling cascades (Fig. 3A). Key enriched pathways included the AGE-RAGE signaling pathway in diabetic complications, Lipid and atherosclerosis, TNF signaling pathway, IL-17 signaling pathway, MAPK signaling pathway, and Toll-like receptor signaling pathway. Furthermore, pathways related to cell survival and metabolism, such as Apoptosis, FoxO signaling pathway, NF-kappa B signaling pathway, and Chemical carcinogenesis (receptor activation and reactive oxygen species) were also significantly enriched (Fig. 3A and Table S9). This indicates that the core targets constitute a central network linking metabolic stress, inflammation, and transcriptional dysregulation in nicotine-induced tumorigenesis. GO functional analysis (Tables S10–S12) revealed that these targets are primarily involved in BP regulating transcription, apoptotic processes, response to hormones, and inflammatory responses. In terms of CC, they were significantly enriched in transcription regulator complexes, RNA polymerase II transcription regulator complexes, and protein complexes. Molecular function analysis highlighted their roles in DNA-binding transcription factor binding, RNA polymerase II-specific DNA-binding, and protease binding. These results suggest that nicotine may drive LUAD progression by modulating gene transcription machinery and altering cell fate decisions.

### GeneMANIA-based functional association network analysis

To capture a more comprehensive landscape of nicotine toxicity, we expanded the network using GeneMANIA-based functional association (GMFA) analysis. The four core targets (CASP3, IL1B, FOS, and JUN) were extended to a network of 39 genes (GMFA-ED) by incorporating functionally associated genes. Comparative KEGG enrichment analysis of the original 12 core targets *versus* the 39 GMFA-ED genes revealed both conserved mechanisms and distinct functional modules (Figs. 3B–3C and Tables S13–S15). Consistently, both gene sets showed significant enrichment in fundamental signaling pathways, including MAPK, TNF, Apoptosis, and IL-17 signaling. Notably, critical hormonal regulations, such as Estrogen, Prolactin, and Relaxin signaling pathways, were also conserved in both sets, indicating that the endocrine-immune axis is central to the core mechanism. Distinctly, the 12 core targets were exclusively enriched in transcriptional and metabolic stress hubs, specifically the NF-kappa B and FoxO signaling pathways, suggesting these targets act as primary drivers of transcriptional dysregulation. In contrast, the expanded GMFA-ED network revealed unique mechanistic insights that were absent in the core set alone. The 39-gene set showed broader involvement in neuro-signaling and addiction-related pathways, such as Dopaminergic synapse, Cocaine addiction, and Amphetamine addiction, as well as specific adaptive immune responses like Th1 and Th2 cell differentiation. Collectively, these findings suggest that while the core targets drive the immediate transcriptional and inflammatory response, the broader functional network encompasses systemic effects involving neuro-addiction mechanisms and complex immune modulation, providing a systemic view of nicotine-induced LUAD pathogenesis.

### Gene set variation enrichment analysis of GMFA-ED

To assess the directional activity of the expanded functional network, we performed GSVA (Fig. 3D). The analysis revealed a distinct regulatory dichotomy in nicotine-associated genomic activities. Positive enrichment was observed in pathways governing cell fate and specific immune responses, notably Apoptosis, IL2-STAT5 signaling, PI3K-AKT signaling, and Interferon Gamma response, suggesting a transcriptional upregulation of these survival and regulatory axes. Conversely, key pro-inflammatory and tumor-suppressive cascades, including TNF-α signaling *via* NF-κB, Inflammatory response, IL6-JAK-STAT3 signaling, and the p53 pathway, displayed negative enrichment scores. This pattern implies that nicotine exposure may drive LUAD progression by simultaneously activating proliferation/survival signals (PI3K-AKT) while suppressing acute inflammatory responses (TNF/IL6) and p53-mediated tumor surveillance.

### Competing endogenous RNA network construction

A ceRNA network was constructed to elucidate regulatory mechanisms underlying identified targets and their interactions. A total of eight, six, three, and 15 miRNAs targeting JUN, CASP3, IL1B, and FOS, respectively, was retrieved from the miRDB (Fig. 3E and Table S16). After removing the duplicates, 31 unique miRNAs were identified. Additionally, lncRNAs associated with these miRNAs were predicted using the ENCORI database and 25 unique lncRNAs were identified after removing duplicates. The ceRNA network showed high values for miR-101, miR-155-5p, and lncRNAs likes, X inactive specific transcript (XIST), small nucleolar RNA host gene 14 (SNHG14), and OIP5 antisense RNA 1(OIP5-AS1) (Fig. 3E); The ceRNA network provides a comprehensive overview of the intricate regulatory relationships between identified targets as well as valuable insights into the molecular mechanisms involved in these diseases.

### Molecular docking

**Figure 4:**
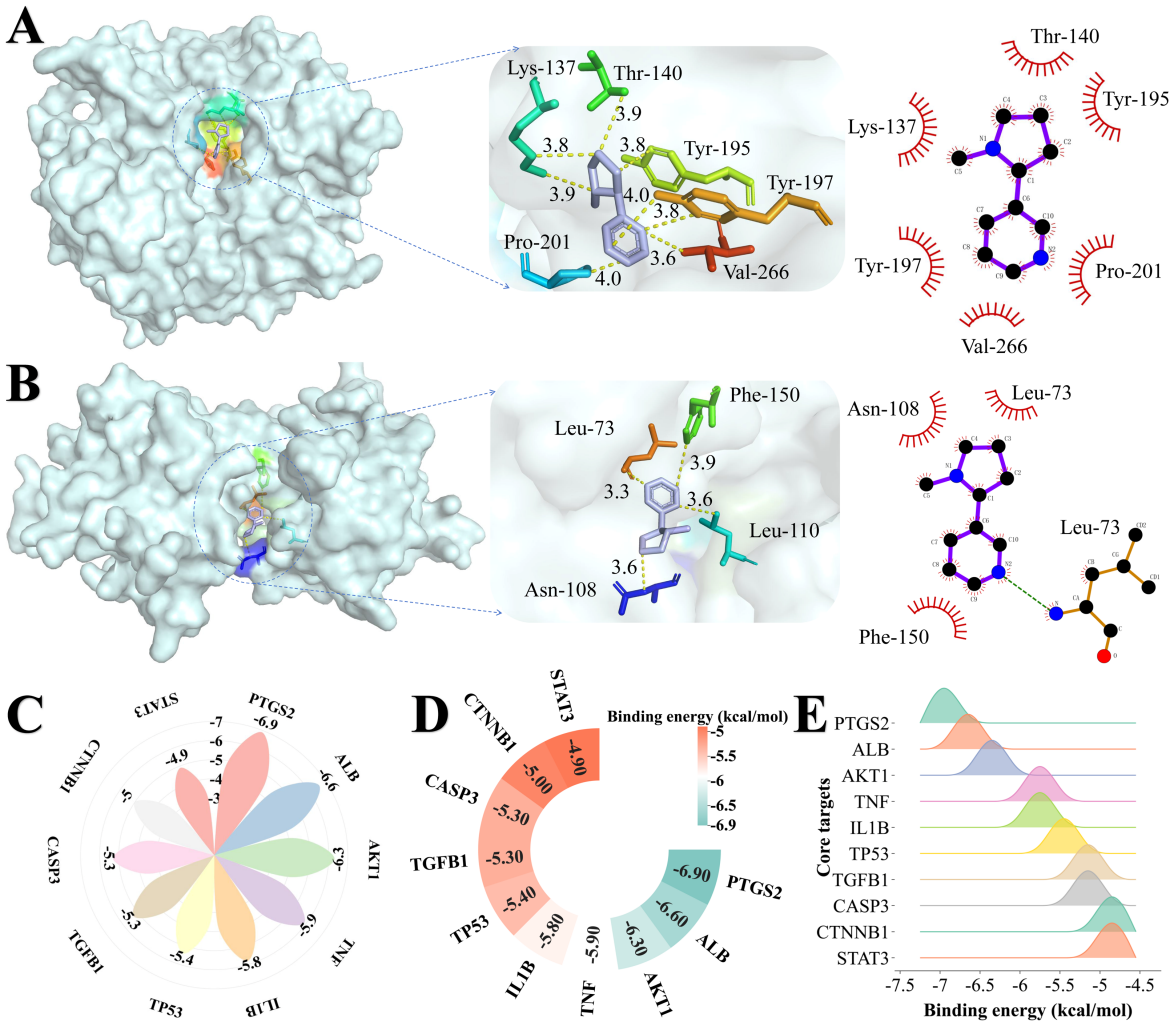
Three- and two-dimensional docking patterns and interactions of nicotine with four core targets. (A) Docking of nicotine with CASP3. The yellow dotted line in the middle is the interaction bond. Light purple represents nicotine and colored parts are amino acids connected to it. (B) Docking of nicotine with IL1B. (C) Rose plot of nicotine docking binding energy with core targets, where larger petals indicate a lower binding energy. (D) Heatmap of nicotine docking binding energy with core targets. (E) Landscape plot of nicotine docking binding energy with core targets.

Molecular docking studies were performed to predict binding affinities and interaction patterns between identified targets and nicotine. Because FOS and JUN showed poor performances in the RCSB PDB database (PBD: 1 FOS) with only C-terminal peptides available, docking could not be performed. Therefore, docking was conducted for the remaining ten targets. The predicted binding energy of CASP3 is −5.3 kcal/mol. The simulation suggests that nicotine may form hydrogen bonds with Tyr197 and Lys137, whereas Thr140, Tyr195, Val266, and Pro201 form hydrophobic interactions (Fig. 4A). Similarly, the Nicotine-IL1B complex showed a binding energy of IL1B is −5.8 kcal/mol, where nicotine is predicted to interact *via* hydrogen bonds with Leu73, whereas Phe-150, Leu-110, and Asn-108 form hydrophobic interactions (Fig. 4B). Additionally, the analysis indicated favorable thermodynamic affinities for PTGS2 (−6.9 kcal/mol), ALB (−6.6 kcal/mol), and AKT1 (−6.3 kcal/mol; Table S17 and Figs. 4C–4E). These computational results propose a structural hypothesis for potential molecular interactions between nicotine and these core targets, providing preliminary insights into mechanisms that warrant further experimental investigation.

### Molecular dynamics simulations

**Figure 5:**
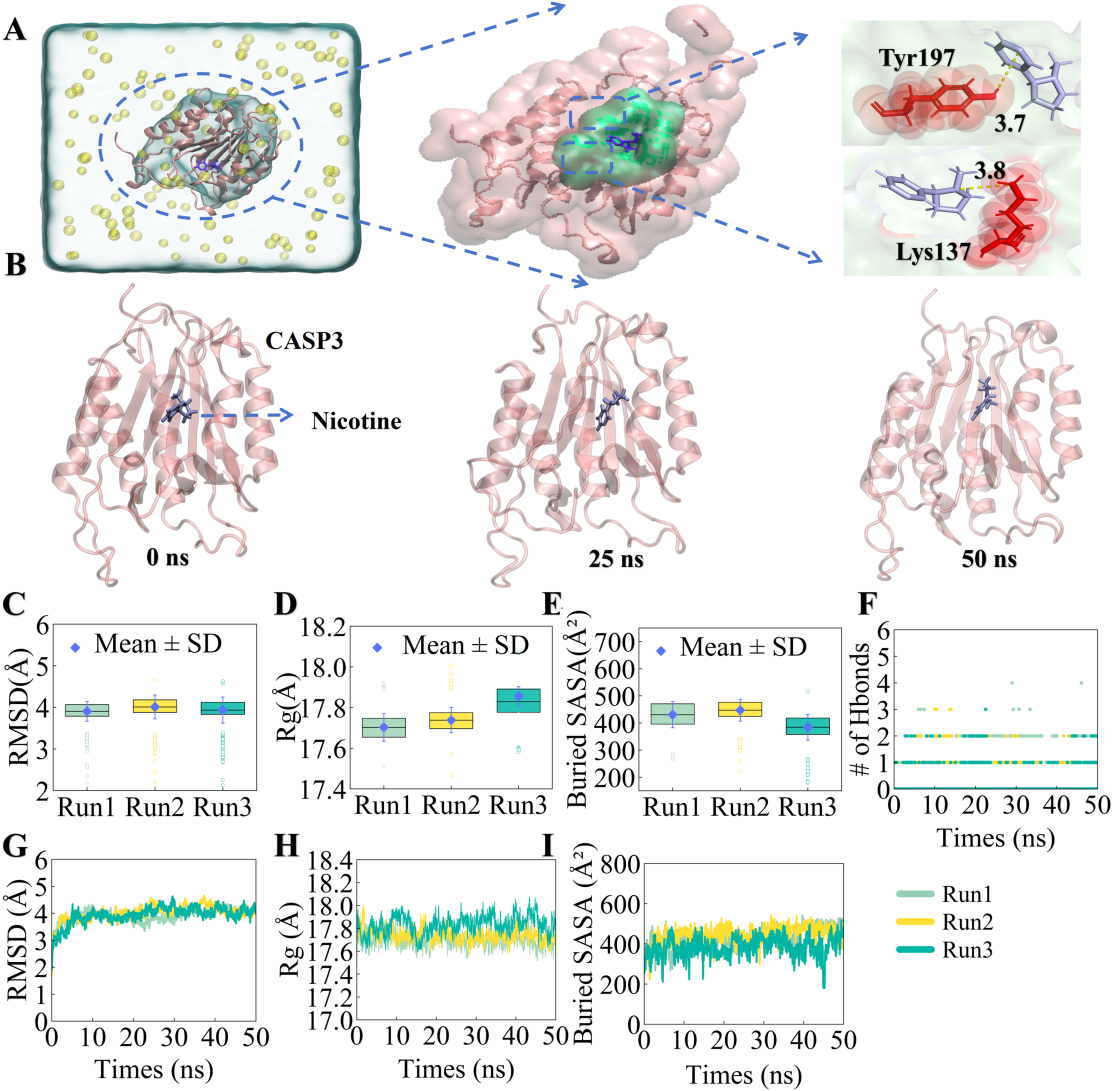
Molecular dynamics simulations of nicotine and CASP3 protein were performed for 50 ns. (A) Nicotine ligands and CASP3 proteins immersed in an ionic water framework. (B) Snapshots of the nicotine–CASP3 complex at 0, 25, and 50 ns, showing the relative stability of nicotine at the binding site during the simulation. (C–E) Quantitative analysis of the system’s stability, displaying the statistical distribution of RMSD, Rg, and Buried SASA across three replicate runs. Data are presented as box plots representing the mean ± SD. (F) Time-evolution of the number of hydrogen bonds formed between nicotine and CASP3 during the simulation. (G–I) Time-dependent dynamic trajectories showing the fluctuations of RMSD, Rg, and Buried SASA over the course of the simulation, confirming the structural stability of the complex.

Because molecular docking cannot account for factors such as protein flexibility, temperature, pressure, and solvent effects, we conducted 50 ns MD simulations to further assess the binding stability of the nicotine-CASP3 complex, which was selected due to its docking affinity and biological relevance (Jia et al., 2016; Wen et al., 2011) (Figs. 5A–5B and Fig. S4).

To rigorously evaluate stability, we first performed a quantitative statistical analysis of key metrics across replicate runs. The RMSD distribution showed that the complex maintained a highly stable binding state, with structural fluctuations confined within a narrow amplitude of 5 Å (Fig. 5C). Similarly, the Rg value reflecting structural compactness exhibited minimal variation, with fluctuations consistently confined within the range of 17.5 to 18.0 Å (Fig. 5D). The Buried SASA analysis further confirmed a robust and consistent contact interface between the ligand and protein (Fig. 5E). Regarding intermolecular interactions, the number of hydrogen bonds fluctuated stably between one and three, providing persistent electrostatic anchoring (Fig. 5F). These statistical findings were visually corroborated by the time-dependent trajectories of RMSD, Rg, and Buried SASA (Figs. 5G–5I), which showed no significant dissociation or unfolding events throughout the simulation. Finally, Residue Interaction Intensity (RII) analysis identified Tyr197 and Lys137 as critical binding residues (Table S18), consistent with the initial docking predictions.

**Figure 6:**
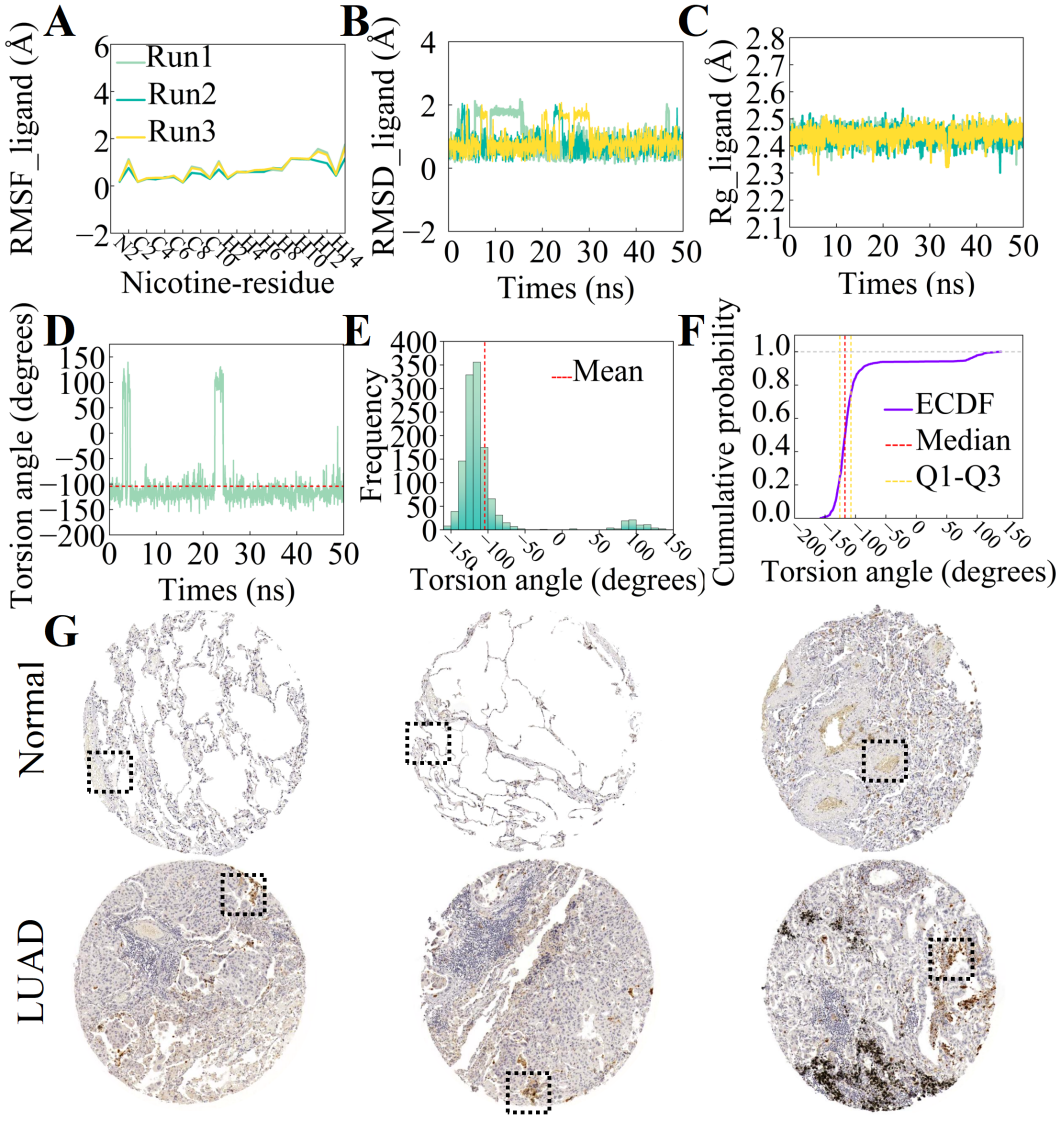
Integrated analysis of nicotine dynamics and CASP3 immunohistochemistry. (A) Root-mean-square fluctuation (RMSF) curves of the nicotine molecule across three independent simulation runs (Run1, Run2, Run3). (B) Root-mean-square deviation (RMSD) curve of the nicotine molecule during the simulation. (C) Radius of gyration (Rg) curve of the nicotine molecule. (D) Variation of the C1-C2-C6-C7 dihedral angle torsion in the nicotine molecule. (E) Frequency distribution plot of the dihedral angle. (F) Cumulative distribution plot of the dihedral angle. (G) Immunohistochemical analysis of CASP3.

Integrated analysis of the molecular dynamics simulation revealed substantial structural stability of nicotine, evidenced by low RMSF (Fig. 6A), an RMSD of ∼2 Å (Fig. 6B), and a stable Rg (2.4−2.5 Å) (Fig. 6C). The dihedral angle underwent an initial adjustment before equilibrating at 25 ns (Fig. 6D). Its distribution, primarily between −130° and 120° with a major at −125° (Figs. 6E and 6F), agrees with the previously reported C1-C2-C6-C7 angle of −126.8° (Arnaud et al., 2007), indicating an approximately perpendicular arrangement of the pyridine and pyrrolidine rings. These findings collectively demonstrate the conformational dynamics and stability of nicotine, offering crucial information for elucidating its biological interactions. IHC staining revealed a marked increase in CASP3 protein expression in LUAD tissues compared to normal lung tissues (Fig. 6G).

## Discussion

**Figure 7:**
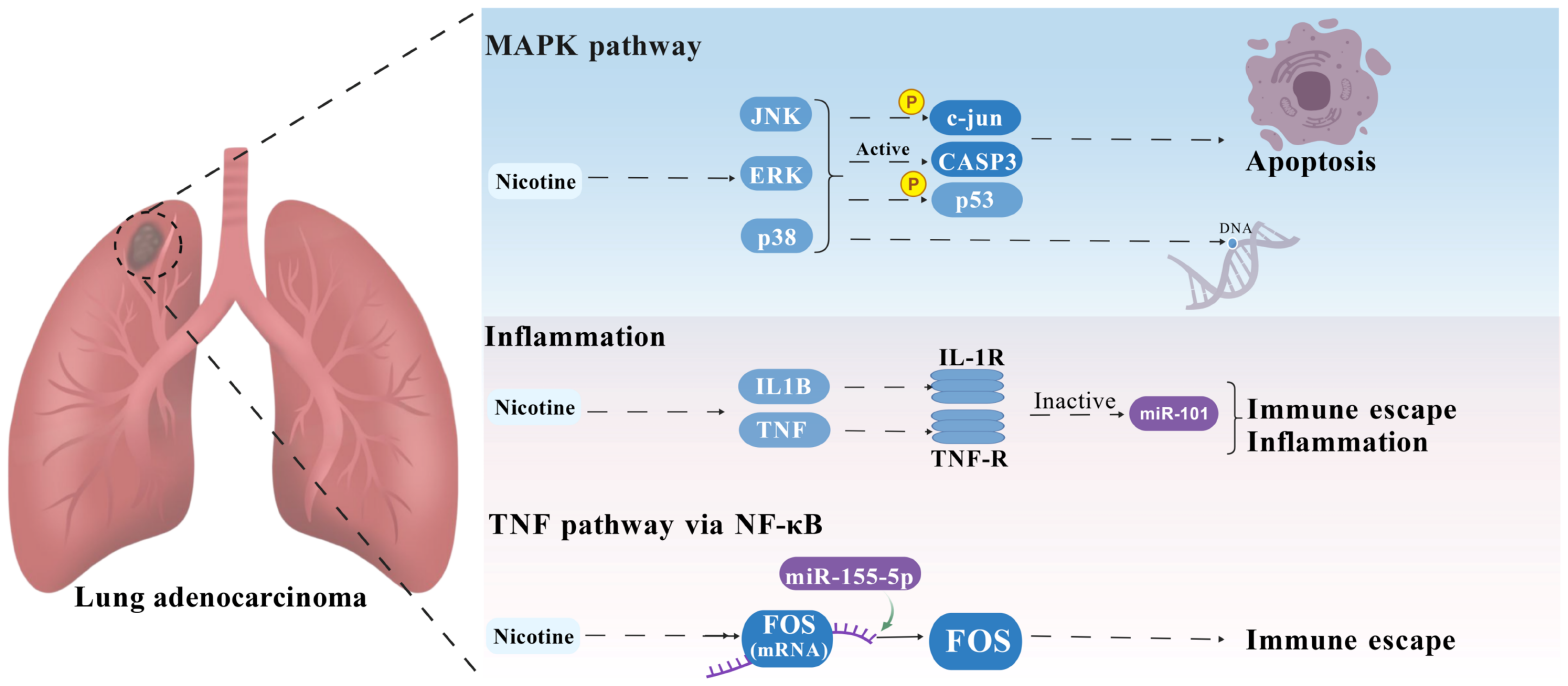
Mechanisms of nicotine-induced lung adenocarcinoma. Nicotine regulates the transcriptional control and immune evasion through TNF signaling *via* NF-κ B, inflammation, apoptosis, and MAPK pathway.

LUAD, a prevalent subtype of NSCLC, causes significant global mortality. Nicotine from cigarettes contributes to environmental contamination and LUAD progression (Murphy, 2021; Thandra et al., 2021). By integrating data from TCGA, CTD, STITCH, GeneCards, and OMIM databases, we identified potential targets of nicotine exposure associated with LUAD. After constructing a PPI network, we identified four core targets, JUN, CASP3, IL1B, and FOS, which have diagnostic significance in nicotine-induced LUAD. In addition, nicotine may promote the progression of LUAD by regulating several biological processes, including the TNF signaling pathway, inflammation, apoptosis, and MAPK pathway, a finding supported by GSVA, Gene Ontology (GO), and Kyoto Encyclopedia of Genes and Genomes (KEGG) enrichment analyses. Within the ceRNA network, miR-101, miR-155-5p, and lncRNAs (XIST, SNHG14, and OIP5-AS1) were identified as potential regulators. Molecular docking and MD simulations further predict the stability of nicotine and core genes. The results of this study provide valuable insights into the molecular mechanisms of nicotine-induced LUAD and highlight the potential of network toxicology (Fig. 7, created with BioGDP.com) and ceRNA network analysis in identifying new therapeutic targets, thus advancing our understanding of the role of nicotine in LUAD and providing prevention and treatment strategies.

MR analysis explored the causal relationships of smoking and nicotine with LUAD (Figs. S1 and S2). Consistent with our findings, researchers previously showed that *in vitro* exposure enhances the expression of PD-L1 and the malignant phenotype of LUAD cells (Lu et al., 2021). By combining the analysis of the network toxicology of nicotine, the main addictive component of cigarettes, we further confirmed the association and gained insight into underlying molecular mechanisms. We identified four key genes essential for LUAD progression (Fig. 2E): JUN, CASP3, IL1B, and FOS. Enrichment analyses showed that these genes are involved in TNF signaling *via* NF-κB, inflammation, apoptosis, and MAPK pathways (Figs. 3D and 4E).

TNF is a key inflammatory cytokine that activates NF-κB but can also induce apoptosis and necroptosis (Cabal-Hierro & Lazo, 2012; Wertz, 2014). IL-1B and TNF are key targets of this pathway (Fig. 3D and Table S14). Previous studies indicate that smoking reduces the generation of IL-1B, IL-2, TNF-α, and IFN-γ through monocytes and decreases IL-6 production *via* Toll-like receptors TLR-2 and TLR-9, mechanisms that are thought to damage respiratory epithelial cells and promoting tumor progression while impairing anti-tumor immunity (Ouyang et al., 2000; Zuo et al., 2019). Consistently, in LUAD cells, active IL-1B binds to the IL-1 receptor, downregulates tumor-suppressive miR-101, and induces IDO1 expression and its metabolite Kyn, processes known to suppress T-cell activity and facilitates immune evasion (Opitz et al., 2020). Specifically, cigarette smoke inhibits TNF-α and interferon gamma (IFN-γ) in natural killer (NK) cells, while nicotine downregulates anti-inflammatory microRNAs and impairs neutrophil function, which may reduce pathogen resistance (Keast & Taylor, 1983; Yamaguchi, 2019).

Inflammation is a well-known driver of lung cancer development and smoking induces persistent lung inflammation and tissue damage, significantly increasing the risk of lung cancer (Engels, 2008). Notably, inflammatory response pathways were significantly enriched and showed a strong negative association with LUAD progression (*P* < 0.05) (Fig. 3D). This finding aligns with experimental evidence showing that that LUAD cells may escape strict homeostatic control by downregulating the expression of TNFAIP3 (also known as A20), a potent anti-inflammatory protein (Breitenecker et al., 2021). Literature suggests that intrinsic loss of A20 in tumor cells results in the hyperactivation of TANK-binding kinase 1 (TBK1) and increased expression and activation of STAT1, leading to elevated PD-L1 levels. This mechanism is proposed to prevent CD8+ Tcell-mediated immune surveillance in both patients and mouse models, potentially enhancing the development of LUAD (Breitenecker et al., 2021; Skoulidis et al., 2018).

Apoptosis refers to caspase-mediated programmed cell death (Chen, Kang & Fu, 2018) and TNF, CASP3, and FOS were identified as targets involved in this pathway (Figs. 3E and 7). Specifically, in the extrinsic apoptotic pathway, the binding of TNF-α, FASL, and TRAIL to their respective receptors triggers the autoproteolytic conversion of procaspase-8 to CASP8, which subsequently activates CASP3, leading to apoptosis. The miRNA let-7a-1 mediates CASP3, which causes cancer cells to develop multidrug resistance and evade apoptosis (Chen, Kang & Fu, 2018; Lin et al., 2013). In LUAD, our network analysis suggests that nicotine and miR-155-5p may regulate cell cycle regulators, such as FOS (Fig. 3E), potentially leading to tumor progression and immune evasion. The results of previous studies align with our predictions. In A549 NSCLC cells, nicotine exposure induces the formation of an ARRB1-E2F1-EP300 complex, which promotes histone and E2F1 acetylation, thereby activating genes related to proliferation and survival. Concurrently, *δ*-opioid receptor signaling triggers ARRB1 nuclear translocation, leading to the upregulation of cell cycle regulators such as CDKN1B and FOS (Dasgupta et al., 2011). Interestingly, ceRNA network analysis predicted that miR-155-5p targets FOS (Fig. 3E). Researchers have observed that the expression levels of miR-155-5p and miR-377-3p are significantly lower in lung cancer cells than in normal human lung epithelial cells, suggesting they may be involved in tumor immune evasion through the PD-L1 axis (Xia et al., 2021).

The MAPK pathway, a key signaling cascade in cellular regulation, comprises three main subfamilies: extracellular signal-regulated kinase (ERK MAPK), JNK/stress-activated protein kinase (SAPK), and MAPK14 (Lee, Rauch & Kolch, 2020). CASP3, TP53, AKT1, and STAT3 were enriched in this pathway (Figs. 3D, 7 and Table S14). Experimental studies in murine lungs demonstrate that nicotine activates JNK, ERK, and p38 MAPK, which upregulate CASP3, FasL, Bax, t-Bid, and cytochrome C; increase the phosphorylation of p53 and Fas; and downregulate bcl-2, thereby inducing nicotine-mediated apoptosis and promoting cell growth in adenocarcinoma cell lines derived from nonsmokers (Kuo et al., 2005; Wen et al., 2011). Mechanistically, nicotine can cause Ca2+ influx into lung cancer cells by binding to α7nAChR and triggering membrane depolarization, which activates voltage-gated Ca2+ channels and subsequently activates the MAPK pathway (Heusch & Maneckjee, 1998). Specifically, in LUAD cells, nicotine-derived nitrosamides (NNK) bind to the α7 nicotinic acetylcholine receptor (α7nAChR), leading to increased expression of sarcoplasmic CD36, which activates Src and the downstream pro-carcinogenic kinases ERK1/2 and AKT, a cascade that ultimately leads to the formation of subcutaneous and pulmonary metastatic tumors (Li et al., 2023). Furthermore, *in vitro* studies showed that α5-nAChR inhibits CASP3 expression, promotes STAT3 phosphorylation, and binds to the CD47 promoter, thereby stimulating nicotine-related LUAD cell apoptosis and immune escape (Jia et al., 2016; Kang et al., 2023).

Based on the ceRNA network, several key molecules associated with nicotine-induced LUAD were identified. Among them, miR-421, miR-4500, miR-5688, and miR-495-3p are recurrent miRNAs that are predicted to play critical roles in nicotine-induced LUAD by targeting genes such as CASP3 and IL1B, suggesting their potential as novel biomarkers (Fig. 3E). Additionally, lncRNA X-inactive specific transcript (XIST) targets multiple miRNAs, including miR-139-5p, miR-524-5p, and miR-520d-5p (Fig. 3E), indicating its potential role in the regulation of nicotine-related signaling pathways. Molecular docking demonstrates that nicotine exhibits a binding affinity for ten key targets (Fig. 4D), supporting the hypothesis that nicotine may promotes LUAD progression through direct or indirect regulation of these targets. Simulations provided atomic-level details of the interactions and conformational changes (Figs. 5A and 6D), indicating stable binding dynamics and suggesting a potential functional role in LUAD development.

In summary, this study integrates MR, network toxicology, and molecular dynamics simulations to elucidate the pathogenesis of nicotine-induced LUAD. The identified core targets and signaling axes provide valuable insights into the molecular etiology and highlight potential biomarkers for early diagnosis. Furthermore, the successful application of this multi-dimensional framework provides a novel methodological approach and technical support for inferring the mechanistic relationships between other environmental pollutants and complex diseases, offering a theoretical foundation for future precision medicine strategies.

### Limitations

Despite the promising findings, several limitations of this study must be acknowledged. First, our analyses rely heavily on public databases (*e.g.*, TCGA, GEO, drug-target repositories), which may contain inherent biases related to sample heterogeneity, sequencing platforms, or data curation updates. Second, although we employed rigorous filtering criteria and high-fidelity simulations (molecular docking and MD simulations) to enhance reliability, these findings remain computational predictions. The complex physiological environment of the human lung, involving dynamic immune interactions and metabolic fluctuations, cannot be fully replicated in silico. Finally, the lack of immediate wet-lab validation (*in vitro* or *in vivo* experiments) in the current study means that the causal roles of the identified targets, while statistically robust, await biological confirmation. Future research should prioritize validating these key molecular mechanisms through animal models and large-scale prospective clinical cohorts.

## Conclusions

In this study, the potential mechanisms underlying nicotine-induced LUAD were explored using an integrated approach involving MR, network toxicology, molecular docking, and MD simulations. While MR analysis supported a causal link between nicotine and LUAD incidence, suggesting a significant role of nicotine in disease progression. By leveraging multiple databases and constructing a PPI network, our network analysis identified 12 key targets with potential diagnostic significance including CASP3 and IL1B. GO, KEGG, and GSVA analyses indicated a multifaceted regulation of immune evasion, inflammation, and cell death through pivotal pathways such as TNF signaling (*via* NF-κB), inflammation, apoptosis, and the MAPK pathway. The ceRNA network further highlights the potential regulatory role of nicotine in LUAD progression *via* ncRNAs. Molecular docking and MD simulations demonstrated favorable binding modes and stability between nicotine and these key targets, supporting their potential as therapeutic targets. These findings propose a theoretical framework for the multilevel mechanisms of nicotine-induced LUAD, introduce new diagnostic and therapeutic targets, and provide a rationale for precise therapies and public health policies aimed at reducing smoking-related diseases. Our study’s findings rely on the validity of the instrumental variable assumptions used in MR analysis, including relevance, independence, and exclusion restriction. Although sensitivity analyses suggest our results are robust, potential biases due to weak associations, unmeasured confounders, or pleiotropic effects cannot be entirely ruled out. Future studies should focus on investigating the long-term effects of nicotine at varying doses and exposure times, as well as its broader impact on animal models and human samples, to fully elucidate its role in LUAD and potential clinical applications.

##  Supplemental Information

10.7717/peerj.21103/supp-1 Supplemental Information 1 Mendelian randomization (MR) analysis confirming the causal link from smoking to lung adenocarcinoma (LUAD) (A) Causality estimation comparing six MR methods. (B) Scatter plot where the slope represents the causal effect. The X-axis represents the SNP effect on smoking, and the Y-axis represents the effect on LUAD. (C) Forest plot of individual SNP effects. (D) Leave-one-out sensitivity analysis demonstrating that no single SNP dominated the causal estimate. (E) Funnel plot verifying the absence of significant asymmetry. Reliability criteria: Heterogeneity was assessed using Cochran’s Q test (P ¿0.05 indicates no heterogeneity), and horizontal pleiotropy was evaluated via the MR-Egger intercept (P ¿0.05 indicates no pleiotropy).

10.7717/peerj.21103/supp-2 Supplemental Information 2 Mendelian randomization (MR) analysis confirming the causal link from nicotine to lung adenocarcinoma (LUAD) (A) Causality estimation comparing five MR methods. (B) Scatter plot where the slope represents the causal effect. The X-axis represents the SNP effect on nicotine, and the Y-axis represents the effect on LUAD. (C) Forest plot of individual SNP effects. (D) Leave-one-out sensitivity analysis demonstrating that no single SNP dominated the causal estimate. (E) Funnel plot verifying the absence of significant asymmetry. Reliability criteria: Heterogeneity was assessed using Cochran’s Q test (P ¿0.05 indicates no heterogeneity), and horizontal pleiotropy was evaluated via the MR-Egger intercept (P ¿0.05 indicates no pleiotropy).

10.7717/peerj.21103/supp-3 Supplemental Information 3 Validation of the Diagnostic Model and Analysis of Nicotine-Specific Mechanisms (A) Differential expression analysis of the core genes in an independent external validation cohort (GSE31210), comparing Normal tissue (green) and LUAD samples (red). Statistical significance is marked (*p¡0.05, **p¡0.01, ****p¡0.0001). (B) LASSO coefficient profiles of the candidate features. (C) Selection of the optimal tuning parameter (lambda) using 10-fold cross-validation to prevent overfitting. (D) Violin plots illustrating the differential expression of representative core targets (CASP3, AKT1, ALB, JUN, TNF) between Nicotine-Exposed (red) and Non-Exposed (blue) LUAD groups. (E) Box plot demonstrating the dose-dependent of CASP3 expression across five smoking exposure gradients (Control, Non-Smoker, Reformed ¿15y, Reformed ≤ 15y, Current Smoker), confirmed by ANOVA. (F) Bar plot ranking the Spearman correlation coefficients between the 12 core genes and smoking dose intensity. CASP3 and TP53 shows a positive correlation with increased exposure, whereas others exhibit negative associations. (G) Correlation heatmap visualizing the regulatory co-expression patterns between Nicotinic Acetylcholine Receptors (nAChRs) and the 12 core targets. Red indicates positive correlation, and blue indicates negative correlation.

10.7717/peerj.21103/supp-4 Supplemental Information 4 Schematic workflow of the integrated computational strategy for identifying core molecular targets Multi-stage funnel screening process: (Step 1–2) Intersection of nicotine- and LUAD-related targets followed by PPI network analysis identified 12 hub genes. (Step 3) Clinical validation prioritized four diagnostic candidates (JUN, FOS, CASP3, IL1B). (Step 4) Structural feasibility assessment served as a critical checkpoint: FOS and JUN were excluded due to incomplete PDB structures (peptide fragments only), and IL1B was discarded due to unstable binding dynamics. (Step 5) CASP3 was identified as the sole robust candidate for final validation via 50 ns molecular dynamics (MD) simulation.

10.7717/peerj.21103/supp-5 Supplemental Information 5 Supplemental Tables

## Additional Information and Declarations

### Data Availability

The following information was supplied regarding data availability: The code is available at Zenodo: - clarozhong. (2026). clarozhong/Rdata-nicotine: Nicotine-code (v1.0.0). Zenodo. https://doi.org/10.5281/zenodo.18715695
The data is available at the Gene Expression Omnibus (GEO): GSE31210
The transcriptomic data for Lung Adenocarcinoma (LUAD) is available at The Cancer Genome Atlas (TCGA). - https://portal.gdc.cancer.gov/
