# Supplementary figures and images for: Nicotine-induced immune escape mechanisms in lung adenocarcinoma: ceRNA network toxicology, and molecular dynamics simulations
Source: PeerJ. 2026 May 4;14:e21103. doi: 10.7717/peerj.21103 (PMC13151930; doi:10.7717/peerj.21103)

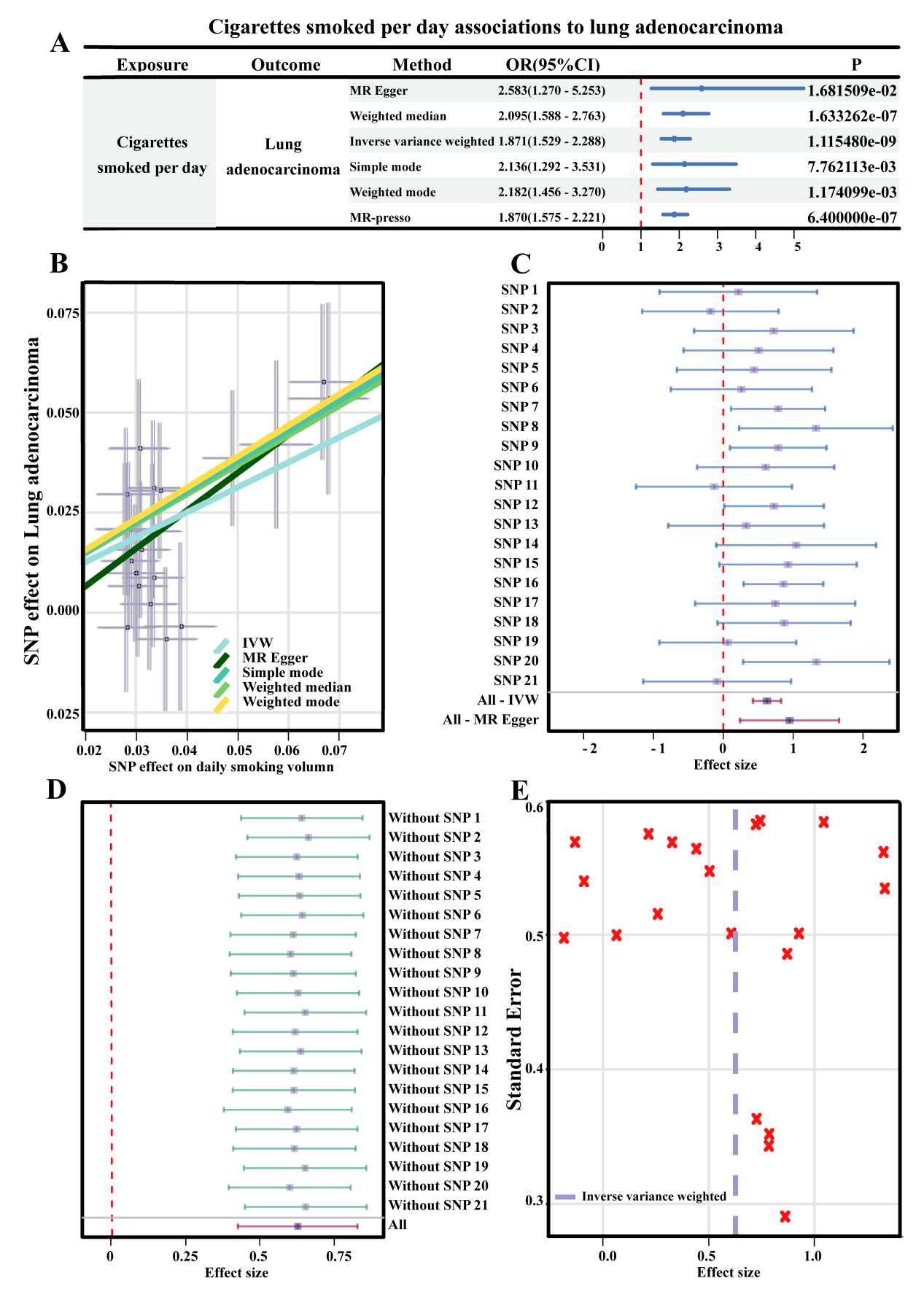

Supplement: Supplemental Information 1 — (A) Causality estimation comparing six MR methods. (B) Scatter plot where the slope represents the causal effect. The X-axis represents the SNP effect on smoking, and the Y-axis represents the effect on LUAD. (C) Forest plot of individual SNP effects. (D) Leave-one-out sensitivity analysis demonstrating that no single SNP dominated the causal estimate. (E) Funnel plot verifying the absence of significant asymmetry. Reliability criteria: Heterogeneity was assessed using Cochran’s Q test (P ¿0.05 indicates no heterogeneity), and horizontal pleiotropy was evaluated via the MR-Egger intercept (P ¿0.05 indicates no pleiotropy). [file peerj-14-21103-s001.png]

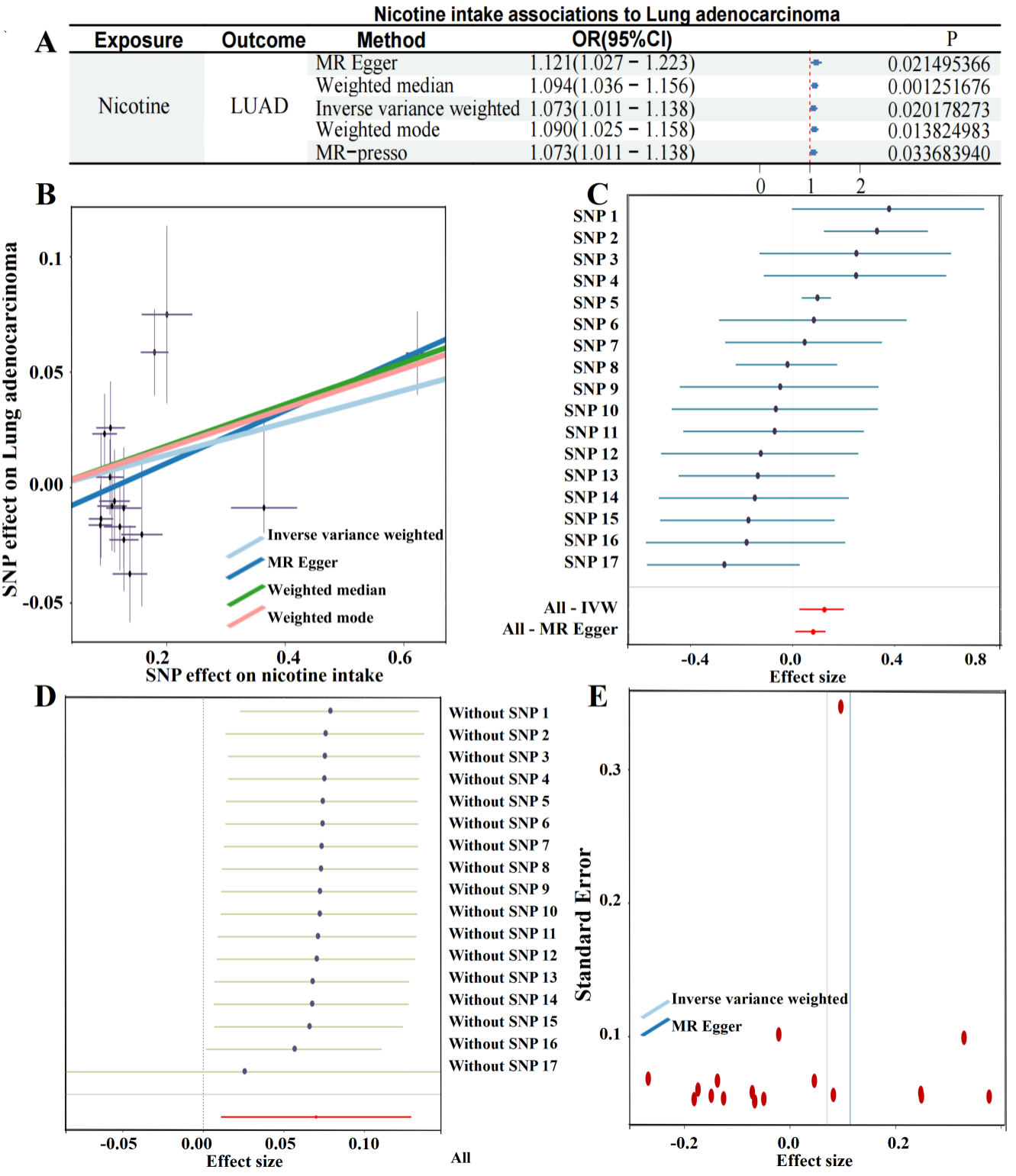

Supplement: Supplemental Information 2 — (A) Causality estimation comparing five MR methods. (B) Scatter plot where the slope represents the causal effect. The X-axis represents the SNP effect on nicotine, and the Y-axis represents the effect on LUAD. (C) Forest plot of individual SNP effects. (D) Leave-one-out sensitivity analysis demonstrating that no single SNP dominated the causal estimate. (E) Funnel plot verifying the absence of significant asymmetry. Reliability criteria: Heterogeneity was assessed using Cochran’s Q test (P ¿0.05 indicates no heterogeneity), and horizontal pleiotropy was evaluated via the MR-Egger intercept (P ¿0.05 indicates no pleiotropy). [file peerj-14-21103-s002.png]

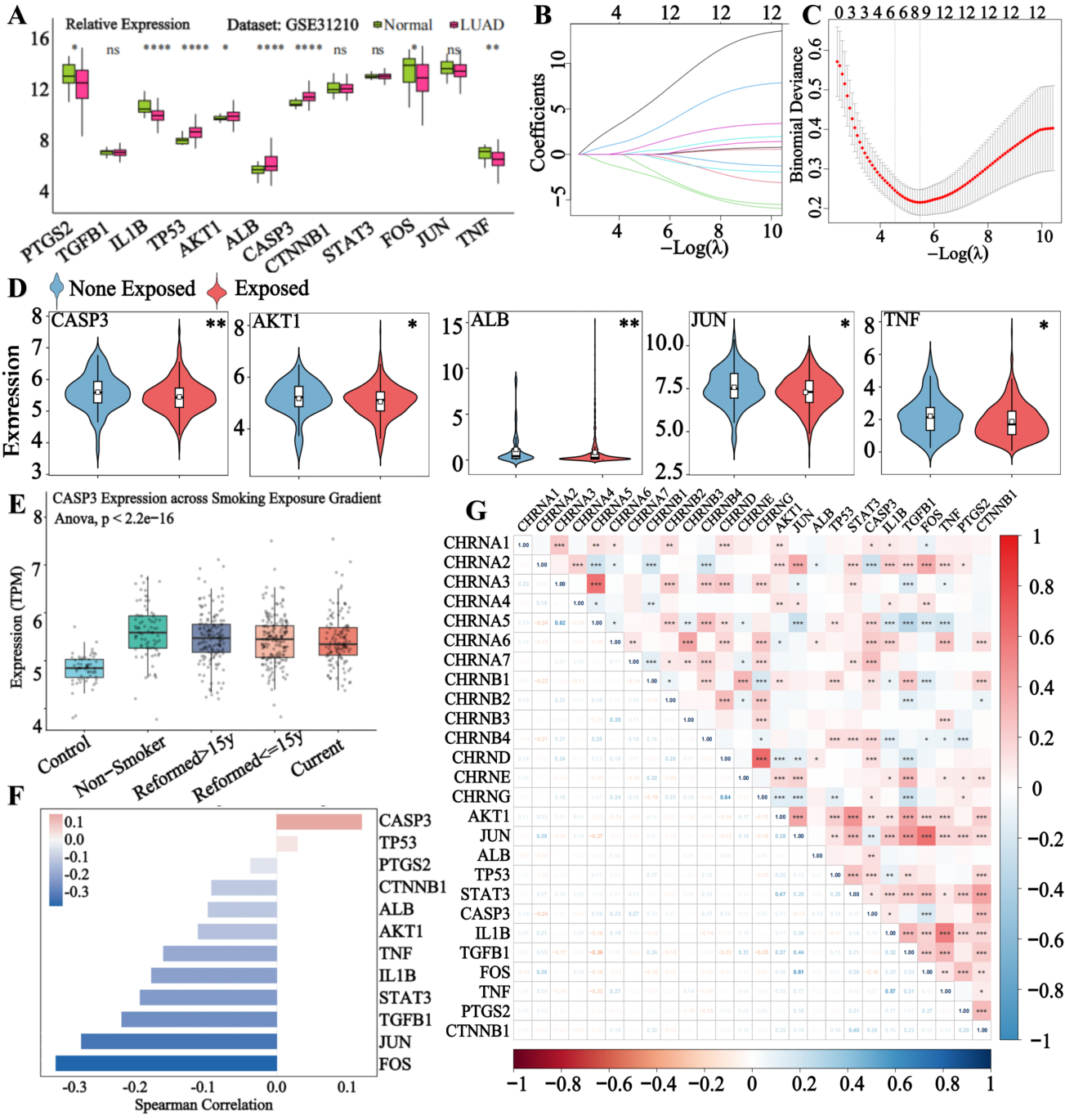

Supplement: Supplemental Information 3 — (A) Differential expression analysis of the core genes in an independent external validation cohort (GSE31210), comparing Normal tissue (green) and LUAD samples (red). Statistical significance is marked (*p¡0.05, **p¡0.01, ****p¡0.0001). (B) LASSO coefficient profiles of the candidate features. (C) Selection of the optimal tuning parameter (lambda) using 10-fold cross-validation to prevent overfitting. (D) Violin plots illustrating the differential expression of representative core targets (CASP3, AKT1, ALB, JUN, TNF) between Nicotine-Exposed (red) and Non-Exposed (blue) LUAD groups. (E) Box plot demonstrating the dose-dependent of CASP3 expression across five smoking exposure gradients (Control, Non-Smoker, Reformed ¿15y, Reformed ≤ 15y, Current Smoker), confirmed by ANOVA. (F) Bar plot ranking the Spearman correlation coefficients between the 12 core genes and smoking dose intensity. CASP3 and TP53 shows a positive correlation with increased exposure, whereas others exhibit negative associations. (G) Correlation heatmap visualizing the regulatory co-expression patterns between Nicotinic Acetylcholine Receptors (nAChRs) and the 12 core targets. Red indicates positive correlation, and blue indicates negative correlation. [file peerj-14-21103-s003.png]

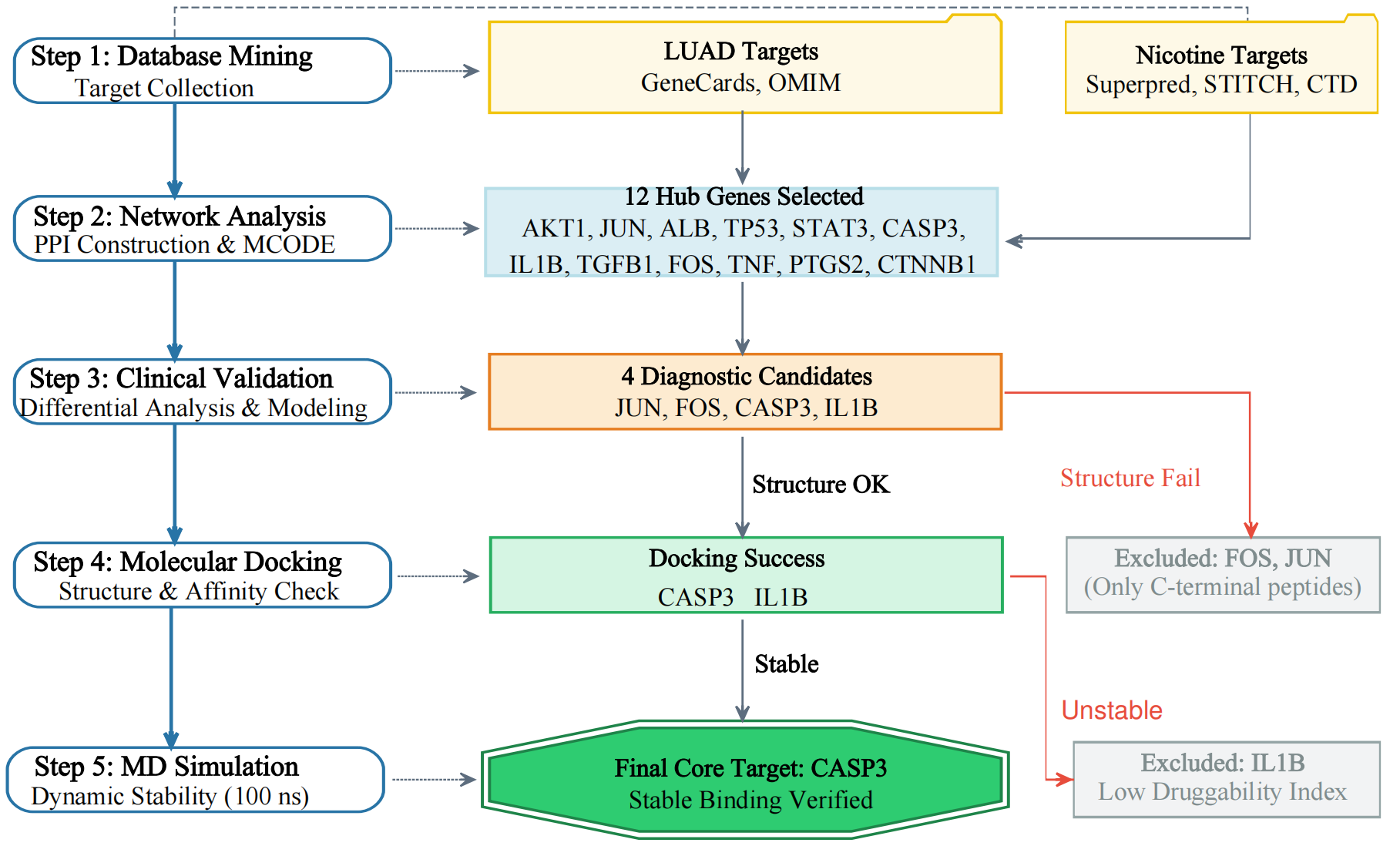

Supplement: Supplemental Information 4 — Multi-stage funnel screening process: (Step 1–2) Intersection of nicotine- and LUAD-related targets followed by PPI network analysis identified 12 hub genes. (Step 3) Clinical validation prioritized four diagnostic candidates (JUN, FOS, CASP3, IL1B). (Step 4) Structural feasibility assessment served as a critical checkpoint: FOS and JUN were excluded due to incomplete PDB structures (peptide fragments only), and IL1B was discarded due to unstable binding dynamics. (Step 5) CASP3 was identified as the sole robust candidate for final validation via 50 ns molecular dynamics (MD) simulation. [file peerj-14-21103-s004.png]
